# Recent Research on the Role of Phytochemicals from Ginseng in Management of Osteosarcoma, Osteoporosis, and Osteoarthritis

**DOI:** 10.3390/nu17111910

**Published:** 2025-06-01

**Authors:** See-Hyoung Park

**Affiliations:** Department of Biological and Chemical Engineering, Hongik University, Sejong 30016, Republic of Korea; shpark74@hongik.ac.kr; Tel.: +82-44-860-2126

**Keywords:** ginseng, ginsenoside, polysaccharide, extract, osteosarcoma, osteoporosis, osteoarthritis

## Abstract

Ginseng phytochemicals have attracted considerable attention for their potential therapeutic applications in bone-related diseases including osteosarcoma, osteoporosis, and osteoarthritis. Recent research has highlighted the promising effects of ginsenosides and polysaccharides from ginseng by studying multi-target effects and combination therapies in osteosarcoma progression. Beyond osteosarcoma, ginseng phytochemicals have been explored for their effects on osteoporosis. Various ginsenosides and ginseng extract were shown to regulate signaling pathways involved in activating osteoblast and inhibiting osteoclast in vitro and in vivo models. Ginseng ginsenosides have also demonstrated potential anti-osteoarthritic properties. Recent studies discussed how ginsenoside reduced inflammation and cartilage degradation as a therapeutic candidate for osteoarthritis management. In this review, we examine the anti-osteosarcoma, anti-osteoporotic, and anti-osteoarthritic activities of ginseng-derived phytochemicals reported in studies published between 2014 and 2024. This review also provides a comprehensive overview of the working mechanisms of these compounds in various model systems. Furthermore, we address the limitations of current research approaches and outline future directions to maximize the therapeutic application of ginseng phytochemicals in the management of bone-related diseases.

## 1. Introduction

### 1.1. Phytochemicals from Ginseng

Ginseng, particularly *Panax ginseng* C.A. Meyer, has been a cornerstone of traditional medicine in Asia for thousands of years. As a member of the Araliaceae family, it is often referred to as an “all-healing” due to its extensive health benefits, largely attributed to active compounds such as ginsenosides [1,2,3]. Ginseng is primarily classified into two types: white ginseng and red ginseng. White ginseng is produced by air-drying fresh roots, whereas red ginseng is produced through a steaming process that modifies its chemical composition to enhance both its therapeutic properties and stability [4,5]. Due to these unique chemical transformations, red ginseng is particularly valued in traditional medicine and is regarded as having superior medicinal efficacy compared to white ginseng. A wide variety of phytochemicals in ginseng have been identified and extensively documented in the literature [6,7,8]. Of them, ginsenosides are the most significant, and over 100 types of ginsenosides have been documented [9,10]. Ginsenosides are broadly categorized into dammarane and oleanane types. The dammarane type is further divided into the protopanaxadiol and protopanaxatriol groups. Some of the well-known ginsenosides in these groups include Rb1, Rb2, Rc, Rd, Re, Rf, Rg1, and Rg3. The oleanane type consists of the oleanolic acid and ocotillol groups, with Ro being a prominent example. In addition to ginsenosides, ginseng contains polysaccharides, phenolic compounds, alkaloids, fatty acids, mineral oils, and amino acids [11,12,13,14,15,16]. Ginsenosides are the most extensively studied among all phytochemicals of ginseng, with Rb1, Rb2, Rc, Rd, Re, Rf, Rg1, and Rg3 being the most abundant. Collectively, these phytochemicals play a pivotal role in mediating the pharmacological effects of ginseng.

### 1.2. Health Benefits and Pharmacological Activities of the Phytochemicals from Ginseng

The primary bioactive components in ginseng are ginsenosides, which are steroidal saponins that contribute to various therapeutic effects. Research has demonstrated that ginsenosides possess anti-oxidative and anti-cancer properties, and can enhance immunity, energy levels, and sexual function, while also combating cardiovascular diseases, diabetes, and neurological disorders [17,18,19,20]. For example, ginsenoside Rg3 has shown promise in killing different types of cancer cells and protecting against DNA damage in normal human cells [21,22]. Ginseng polysaccharides are potential therapeutic agents for various health conditions, including cancer, immune disorders, and metabolic diseases, due to their multifaceted pharmacological activities [23]. A recent study reported that ginseng polysaccharides enhance the production of complement component 4, a core component of the complement system, by promoting its gene transcription via specific promoter regions [24]. In addition, the ginseng berry polysaccharide portion has been shown to suppress cancer growth by activating natural killer cells [25]. Phenolic compounds from ginseng offer numerous health benefits due to their potent anti-oxidant and anti-inflammatory properties [18]. These compounds, including phenolic acids and flavonoids, have been shown to protect against oxidative stress and reduce the risk of chronic diseases such as cancer, cardiovascular disease, and neurodegenerative disorders [1]. Research demonstrated that the total phenol content in ginseng fruit and leaves is higher than in ginseng roots, with chlorogenic acid, gentisic acid, p- and m-coumaric acid, and rutin being the major phenolic compounds found in ginseng [26]. Additionally, steaming ginseng can increase the content and bioavailability of phenolic compounds, potentially enhancing their health-promoting effects [1]. Overall, ginseng is a powerful herbal remedy with a wide range of health benefits, largely due to its diverse phytochemicals, which work synergistically to produce pharmacological effects. Various health benefits of the phytochemicals from ginseng properties could make it a valuable supplement for promoting the well-being of the human body.

### 1.3. Recent Research on Phytochemicals to Manage Osteosarcoma

Osteosarcoma is the most common primary bone cancer, typically affecting children and young adults, with a peak incidence during adolescent growth spurts [27]. It usually develops in the metaphyseal regions of long bones, particularly around the knee, hip, and shoulder, and is characterized by the production of immature bone or osteoid tissue by malignant cells [28]. Common symptoms include localized pain, swelling, and limited range of motion, with diagnosis typically involving imaging studies (X-rays, MRI, CT scans) and biopsy [29]. Although amputation may be necessary in some cases, treatment usually involves a combination of chemotherapy and surgery, with limb-salvage procedures often possible [30]. The prognosis for osteosarcoma has improved significantly over the past few decades, with survival rates around 70% for localized disease, but outcomes are poorer for metastatic or recurrent cases [27]. Phytochemicals show promise as complementary approaches for managing various types of cancer, either alone or in combination with conventional therapies [31]. In fact, there are lots of recent studies on phytochemicals for managing osteosarcoma. A study identified 28 metabolites from soursop leaf extract that showed potential anti-cancer effects against osteosarcoma [32]. Three compounds (2′-hydroxy-5′-methyl chalcone, linoleic acid, and annonacin) showed good binding affinity to platelet-derived growth factor receptor α, a kind of osteosarcoma target protein. The leaf extract exhibited cytotoxicity against MG-63 osteosarcoma cells in vitro. Some phytochemicals can help restore sensitivity of cancer cells to chemotherapy drugs. A study found that combining capsaicin with cisplatin had synergistic inhibitory effects on osteosarcoma cells in culture and in xenografts [33]. The combination induced apoptosis, cell cycle arrest, and inhibited cell invasion more effectively than either compound alone. In addition, compounds such as sulforaphane, carvacrol, thymoquinone, and ursolic acid have shown anti-cancer effects through various mechanisms including cell cycle arrest, apoptosis induction, and inhibition of key signaling pathways [34,35,36,37]. There is continued interest in identifying and evaluating novel plant-derived compounds for their potential in osteosarcoma treatment. More studies are needed to fully elucidate mechanisms of action and optimal dose and combinations for clinical use.

### 1.4. Recent Research on Phytochemicals to Manage Osteoporosis

Osteoporosis is a systemic skeletal disorder characterized by low bone mass and microarchitectural deterioration of bone tissue, leading to increased bone fragility and susceptibility to fractures [38]. It is often called a “silent” disease because it typically doesn’t cause symptoms until a fracture occurs, most commonly affecting the hip, spine, and wrist [39]. The condition develops when bone breakdown exceeds bone formation, resulting in a loss of bone mineral density and changes in bone structure [40]. Osteoporosis is more prevalent in women, especially after menopause, and in individuals over 50 years old [41]. Diagnosis is usually made through bone mineral density (BMD) scans using dual-energy X-ray absorptiometry which measures BMD at the hip and spine [41]. The standard therapy for osteoporosis typically aims to slow or stop bone loss through a combination of lifestyle changes, proper nutrition, and medications. Bisphosphonates are generally considered the first-line treatment for most patients with osteoporosis, especially those at high risk of fracture. These medications work by slowing bone breakdown and have been shown to reduce the risk of fractures by 30–70%, depending on the specific bone site [42]. Recent research has focused on specific plants such as green tea, rosehip berries, ginger root, turmeric root, and pomegranate peel and their potential in osteoporosis management [43]. For instance, a recent study examined the relationship between phytochemical-rich food intake and osteoporosis incidence in pre- and postmenopausal women. The research found that postmenopausal women in the highest phytochemical index quartile had a 16% lower risk of osteoporosis compared to those in the lowest quartile [44]. This suggests that consuming foods rich in phytochemicals may have a protective effect against osteoporosis in postmenopausal women. Also, several phytochemicals have shown promising results in maintaining bone health. Genistein from *Sophora japonica* has demonstrated anti-osteoporosis effects similar to soybean genistein [45,46]. Sophoricoside increased bone mechanical strength and osteogenic biochemical markers in ovariectomized rats [46]. Kurarinone, 8-prenylkaempferol, and (2S)-2′-methoxykurarinone have shown potential in preventing osteoclastic bone resorption and promoting bone health [47]. These phytochemicals work through various mechanisms, such as promoting mesenchymal stem cell differentiation, improving osteoblast proliferation, and suppressing osteoclast formation [43]. While these findings are promising, more research is needed to fully understand the efficacy of phytochemicals in osteoporosis management and to develop targeted interventions. Future studies should focus on validating these results using biochemical data and exploring the potential of combining phytochemicals with conventional treatments for enhanced efficacy in managing osteoporosis.

### 1.5. Recent Research on Phytochemicals to Manage Osteoarthritis

Osteoarthritis is a degenerative joint disease characterized by the breakdown of cartilage and other joint tissues [48]. It is the most common form of arthritis and primarily affects older adults, though it can also occur in younger individuals due to joint injuries or genetic factors [48]. Osteoarthritis typically affects weight-bearing joints such as the knees, hips, and spine, but it can also impact the hands, neck, and lower back [49]. The condition is marked by joint pain, stiffness (especially after periods of inactivity), swelling, and loss of flexibility [49]. Risk factors include age, obesity, previous joint injuries, repetitive stress on joints, genetic predisposition, and sex (with women being more susceptible) [50]. Various treatments can help manage symptoms and improve quality of life. For example, non-steroidal anti-inflammatory drugs (NSAIDs) may be used to control pain [51]. In severe cases, joint replacement surgery may be considered to alleviate pain and restore function [52]. Recent research has increasingly focused on the role of phytochemicals derived from traditional medicinal plants in managing osteoarthritis. These natural compounds found in fruits, vegetables, and herbs, have been recognized for their anti-inflammatory and anti-oxidant properties, making them potential alternatives to conventional pharmacological treatments [53]. The interest in phytochemicals stems from their accessibility, low cost, and the growing desire for complementary and alternative medicine options, particularly for individuals seeking to mitigate the side effects associated with long-term use of NSAIDs and other medications [54]. For instance, a recent study demonstrated that oral administration of *Arnica montana* flower methanol extract reduced clinical signals of osteoarthritis and improved the histological status of hind limb joints in a collagen-induced arthritis rat model [55]. Mechanistically, *Arnica montana* flower methanol extract-treated rats showed lower levels of inflammatory mediators such as nitric oxide and TNF-α. They analyzed the phenolic and flavonoid compounds in *Arnica montana* flower methanol extract by GC/MS and suggested that the therapeutic effects could be due to their combined actions. Another study investigated the protective effects of curcumin on monosodium iodoacetate-induced osteoarthritis rat model [56]. Curcumin treatment significantly reduced joint diameter, improved Mankin’s score, and increased paw withdrawal threshold in osteoarthritis rats. The expression of inflammatory biomarkers MyD88, p-IκBα, NF-κB, IL-6, IL-1β, and TNF-α in synovial fluid was lower in the curcumin-treated group compared to osteoarthritis rat groups. While phytochemicals show promise in treating osteoarthritis, there are several limitations to their use such as lack of rigorous clinical trials and poor understanding of mechanisms. By addressing these limitations, researchers could aim to harness the full potential of phytochemicals as complementary and alternative agents for osteoarthritis treatment, potentially offering more effective and safer options for patients in the future.

### 1.6. Outline of Review

This review focuses on the current understanding and future directions in phytochemicals from ginseng in managing osteosarcoma, osteoporosis, and osteoarthritis. This review deals with main ginseng phytochemicals such as ginsenosides, polysaccharides, phenolic compounds, and alkaloids and emphasizes potential therapeutic targets at various stages of osteosarcoma, osteoporosis, and osteoarthritis progression. By understanding the key signaling pathways and molecules involved in the disease’s onset and development, researchers aim to pinpoint new targets for drug development based on ginseng. This review aims to inform and influence the development of future treatment strategies for osteosarcoma, osteoporosis, and osteoarthritis, providing a comprehensive overview of recent knowledge and emerging trends and serving as a valuable reference for researchers and clinicians in the field.

## 2. Phytochemicals from Ginseng in Management of Osteosarcoma

### 2.1. Ginsenosides

Ginsenosides from *Panax ginseng* have shown promising potential in the management and treatment of various kinds of cancers including osteosarcoma through their pharmacological activities related to cancer cell signaling pathways [57].

Firstly, specific kinds of ginsenosides induce apoptosis in osteosarcoma. Research revealed that ginsenoside Rf triggered G2/M phase cell cycle arrest and promoted apoptosis in MG63 cells via the mitochondrial-mediated pathway [58]. MTT assay result showed the highest cell toxicity of Rf treatment in MG63 cells with 11 μM for 24 h. Ginsenoside Rg3 was reported to induce DNA damage in human osteosarcoma cells, leading to cell cycle arrest and apoptosis [22]. MG63 cell was the most sensitive osteosarcoma cell line with about 150 μM of IC50 value in MTT assay for 24 h incubation. This genotoxic effect was evident through increased DNA single-strand breaks and the formation of γH2AX foci. Another study demonstrated that ginsenoside Rg3 suppressed the proliferation of osteosarcoma cells and induced apoptosis through various signaling pathways, including the PI3K/Akt/mTOR pathway [59]. Interestingly, they showed a relatively low IC50 value for MG3 cells (about 2.5 μM) in CCK8 assay for 24 h incubation. Furthermore, Rg3 treatment was shown to inhibit osteosarcoma development in the MG63 cell xenograft mouse model [60]. Tumor volume and weight in the Rg3 group (20 mg/kg) were reduced up to about 50% for 21 days compared to the control group. They also suggested that the anti-osteosarcoma activity of Rg3 was associated with the circ_0003074/miR-516b-5p/KPNA4 axis. Another study reported that Rg3 inhibited the migration and invasion of osteosarcoma cells via regulation of the Wnt/β-Catenin and EMT signaling pathway [61]. In addition to Rg3, Rg5 was reported to trigger apoptosis by regulating the PI3K/Akt/mTORC1 signaling pathway and activating the LC3-mediated autophagy pathway [62]. Rg5 treatment showed 0.64 μM of the IC50 value for 24 h and a relatively stronger anti-proliferative effect against MG63 cells compared to Rg3. The PI3K/Akt signaling pathway was identified as one of the related signaling pathways that Rg5 might regulate in osteosarcoma treatment [63]. Also, they found PIK3CA, SRC, TP53, MAPK1, EGFR, and VEGFA as target proteins using network pharmacology and molecular docking technology. Anti-osteosarcoma activity of Rh2, another kind of ginsenosides, was reported to impede the growth and migration of U2OS cells [64]. MTT assay results showed that the IC50 value of Rh2 for 48 h was about 8 μM that was slightly higher than that of cisplatin. Rh2 induced apoptosis by regulating MAPK, PI3K/Akt/mTOR, and NF-κB signaling pathways in U2OS. A bioinformatic approach explored how a treatment of Rh2 might work against osteosarcoma by showing the differences between primary and metastatic osteosarcoma at the genetic level [65]. They found that the MAPK and PI3K/Akt/mTOR pathways could play an important role in the metastasis of osteosarcoma, which was targeted by Rh2. Ginsenoside CK was also reported to inhibit the growth, migration and invasion, and survival of human osteosarcoma cells by triggering apoptosis and interfering with the PI3K/mTOR/p70S6K1 signaling pathway [66]. The IC50 value for 72 h was observed around 20 µM in MG63 cells. Western blotting analysis showed that CK treatment regulated PI3K/mTOR/p70S6K1 signaling pathway in MG63 cells, which was enhanced synergistically by RAD001 treatment.

Secondly, ginsenoside stimulates the anti-osteosarcoma activity of other compounds or chemotherapies. A recent study demonstrated that Rg1 enhanced the anti-osteosarcoma activity of Timosaponin AIII, a kind of spirostanol saponin from *Anemarrhena asphodeloides* [67]. The single treatment of Rg1 (250 μM) or Timosaponin AIII (6 μM) did not induce cytotoxic effects in Mg63 cells but the combination treatment of Rg1 (250 μM) and Timosaponin AIII (6 μM) reduced cell viability up to 87% compared to the control. Their combinatorial treatment of them suppressed the MAPK signaling pathway, caused apoptosis, and inhibited the migration of MG63 cells. In another study, the same group reported that Rb1 (250 μM) and Rc (250 μM) had synergistic anti-osteosarcoma activities with Timosaponin AIII (6 μM) in MG63 cells [68]. The synergistic anti-osteosarcoma activity of Rg3 and doxorubicin was also investigated in vitro and in vivo model of osteosarcoma by regulating mTOR/HIF-1α/VEGF and EMT signaling pathways [69]. Colony formation assay result demonstrated that both treatments of Rg3 (80 μg/mL) and doxorubicin (0.3125 μg/mL) in 143B cells caused the synergistic reduction of colony number (almost 0), whereas the colony number of the control, Rg3 only, and doxorubicin only was 430, 287, and 189, respectively. In the 143B xenograft mouse model, the tumor volume of the Rg3 group (20 mg/kg) plus doxorubicin (2 mg/kg) was reduced up to about 30% for 21 days compared to the control group. Compound K (CK), another kind of ginsenoside, enhanced the susceptibility of 143B osteosarcoma cells to cisplatin through induction of apoptosis via mitochondria-mediated pathways such as cytochrome C release [70]. MTT assay results for 72 h showed that CK treatment (10 μM) with cisplatin (5 µM) inhibited 143B cell viability up to about 28% compared to the control. In the xenograft (143B cells) mouse model, tumor volume in the CK and cisplatin administration group (10 mg/kg and 10 mg/kg) were reduced up to about 30% for 28 days compared to the control group. (20S)-protopanaxatriol was reported to enhance MG63 and U2OS cell sensitivity to ferroptosis-inducing chemicals such as RSL3, ML162, and IKE by increasing ACSL4 expression, a key enzyme that promotes lipid peroxidation [71]. The combination of (20S)- protopanaxatriol (0.5 µM) and RSL3 (1 µM) significantly reduced MG63 cell viability up to about 50% compared to the control. Interestingly, (20S)-protopanaxatriol from 0.1 to 100 µM did not affect cell viability. The combined treatment with (20S)-protopanaxatriol and IKE (20 mg/kg and 20 mg/kg) for 28 days suppressed osteosarcoma xenograft volume (74.4% reduction) in vivo compared to the control group.

### 2.2. Polysaccharides

Ginseng polysaccharides have shown promising anti-cancer effects in a lot of studies [11,23,72]. Between 2014 and 2024, there was only one report on the anti-osteosarcoma activity of ginseng polysaccharide [73]. According to the author, polysaccharides with approximately 8 kDa molecular weight from ginseng significantly suppressed the viability of MG63 cells in a dose- and time-dependent manner. IC50 value of polysaccharide was observed around 20 µM in MG63 cells for 24 h. Interestingly, polysaccharides with γ-ray radiation markedly suppressed the colony formation in MG63 cells compared to radiation alone treatment. Polysaccharides were found to induce apoptosis and autophagy, which was associated with a decrease in the phosphorylation of p38 MAPK and Akt in MG63 cells.

### 2.3. Summary

As shown in Table 1, recent studies indicate that ginsenosides could be used as single agents or complementary elements in the treatment of osteosarcoma by offering potential therapeutic options or enhancing the efficacy of conventional therapies. However, there was no specific information about the anti-osteosarcoma effects of different types of phytochemicals such as alkaloids or peptides from ginseng based on the searched publication from 2014 to 2024. The research focused mainly on ginsenosides and polysaccharides, in relation to osteosarcoma. We could find two specific studies dealing with the anti-osteosarcoma activities of ginsenosides. Lu et al. developed nanoparticles with thicknesses from 2 to 3 nm by combining graphene oxide with indocyanine green, folic acid, polyethylene glycol, and Rg3 [74]. They investigated the therapeutic potential of these nanoparticles in conjunction with photodynamic therapy for treating osteosarcoma in vitro and in vivo models.

When used as a single treatment, the IC50 value of ginsenosides was arranged from 0.64 to 150 µM, which was evaluated by cell viability approaches such as MTT, CCK8, EDU, and colony formation assay. The treatment time with ginsenosides varied among studies and most experiments were performed for 24 h. MG63, U2OS, and 143B osteosarcoma cells were adapted as in vitro model systems. The main target of ginsenosides and polysaccharides was demonstrated to be the PI3K/Akt and MAPK signaling pathway. Also, ginsenosides and polysaccharides showed anti-osteosarcoma activities such as the promotion of apoptosis and inhibition of migration. Just one study evaluated the anti-osteosarcoma activities of Rg3 in a xenograft mouse model using MG63 cells. In the case of combination treatment, five studies showed that ginsenosides enhanced the anti-osteosarcoma activity of Timosaponin AIII, cisplatin, doxorubicin, and RSL3. While these findings are promising, it is important to note that most of the research has been conducted in vitro on cell lines. Further studies, especially clinical trials, are needed to fully establish the efficacy and safety of ginsenosides or polysaccharides from ginseng as a treatment for osteosarcoma in humans.

## 3. Phytochemicals from Ginseng in Management of Osteoporosis

### 3.1. Ginsenosides

Various ginsenosides, including Rb1, Rb2, Rc, Re, Rg1, Rg3, Rg5, Rh1, Rk1, and CK, found in ginseng, have shown promising effects in the treatment of osteoporosis [75,76]. Ginsenosides have been reported to activate the differentiation of osteoblasts and inhibit the function of osteoclasts by regulating bone formation-related signaling pathways and markers in vitro and in vivo models [75].

Zhu et al. reported that ginsenoside Rb1 attenuated aluminum chloride (AlCl3)-induced impairment in osteoblasts isolated from the skull of the rats [77]. CCK8 assay showed that the viability of osteoblast was decreased by AlCl3 (0.126 mg/mL) to 10% but increased by AlCl3 (0.126 mg/mL) plus ginsenoside Rb1 (0.0145 mg/mL) to 145% compared to the control. In addition, Rb1 treatment alleviated the reduced mRNA expression level of TGF-β1, BMP-2, IGF-I, and Cbfα1 and the increased ROS concentration caused by AlCl3 in osteoblast. Another study demonstrated the anti-osteoporosis activity of Rb1 in dexamethasone (DEX)-induced osteoporosis rat model by regulating aryl hydrocarbon receptor (AHR)/proline/arginine-rich end leucine-rich repeat protein (PRELP)/NF-κB signaling pathway [78]. Bone density (about 170 mg/cm^3^) in DEX-induced osteoporosis rats was lower than that of the control rats (300 mg/cm^3^). Remarkably increased bone density (280 mg/cm^3^) was observed in DEX-induced osteoporosis rats administrated with Rb1 (6 mg/kg) for 12 weeks, which was about the similar effect by 15 μg/kg of 17 β-estradiol (E2). They also suggested that the upregulation of AHR by Rb1 could activate the expression of PRELP leading to inhibition of NF-κB pathway, which underlays the anti-osteoporotic impact of Rb1. However, another study indicated that Rb1 did not prevent bone loss in the ovariectomized (OVX) osteoporosis rat model [79]. Authors demonstrated that Rb1 (10 μM) with osteogenic medium increased alkaline phosphatase activity (ALP) up to about 140% in mesenchymal stem cells from rat bone marrow compared to osteogenic medium. Also, Rb1 increased mineralization and the expression of osteogenic proteins, such as osteopontin, osteoprotegerin, ALP, and Runt-related transcription factor 2 (Runx2). However, administration of Rb1 (6 mg/kg) for 12 weeks could not recover the bone loss of the lumbar vertebra and femur that was decreased significantly in OVX rats. Thus, it is still necessary to acknowledge that the results of in vitro experiments may not always align with those of in vivo experiments and there might be differences in experimental results based on in vivo models.

Huang et al. investigated the anti-osteoporosis activity of ginsenoside Rb2 in the MC3T3-E1 cell model and in the ovariectomized osteoporosis mouse model [80]. Rb2 (10 μM) treatment for 48 h nearly restored the ALP activity in MC3T3-E1 cells damaged by H2O2 treatment. The administration of Rb2 (18.5 μmol/kg) for 12 weeks in the ovariectomized mouse almost recovered bone mass and structure of the distal femur. Mechanistically, Rb2 reduced H2O2-induced oxidative stress and the expression of bone-resorbing factors such as receptor activators of NF-κB ligand (RANKL) and IL-6. Another study demonstrated that Rb2 suppressed osteoclast differentiation of RAW264.7 cells by regulating NF-κB and signal transducer and activator of transcription protein 3 (STAT3) signaling pathway [81]. After promoting RAW264.7 cells with RANKL, Rb2 (10 μM) treatment effectively reduced the formation of tartrate-resistant acid phosphatase (TRAP)-positive multinucleated cells by up to 30% compared to the control. Recently, Rb2 has been reported to show anti-osteoporosis activity in a ketogenic-diet-induced osteoporosis mouse model [82]. Microstructures of cancellous bone in the distal femur were degenerated in the ketogenic-diet-induced osteoporosis mouse, which was analyzed by micro-CT. The authors demonstrated that compared to the control group, bone mineral density (BMD) was increased by 70.7% in the ketogenic-diet-induced osteoporosis mouse administrated with Rb2 (20 mg/kg) in 12 weeks.

Ginsenoside Rc has been reported to display anti-osteoporosis activity and the underlying mechanisms in vitro and in vivo [83]. Authors showed that Rc (500 μM) treatment for 14 days increased the ALP activity in MC3T3-E1 cells up to about 260% compared to the control. They also proved that administration of Rc (50 mg/mL) for 3 months recovered the reduced BMD parameter (14%) in the OVX mouse group, which had about the same effect as 1 mg/kg of E2. Rc enhanced MC3T3-E1 cell development by activating the Wnt/β-catenin signaling cascade. Wang et al. published the anti-osteoporosis result using Rc in the primary osteoblasts cells from human skulls and OVX rats [84]. ALP activity in the primary osteoblasts cell was increased up to 200% by Rc (10 μM) treatment. The BMD parameter in OVX rats was apparently reduced to the control group, while Rc (20 mg/mL) or E2 (2 μg/kg) administration for 4 weeks rescued the decreased BDM parameter of femurs up to 122 and 125% in the OVX rats. In addition, Western blotting results revealed that Rc administration upregulated the TGF-β/Smad signaling pathway in femur tissues of OVX rats.

Ginsenoside Re promoted osteoblast differentiation in MC3T3-E1 cells and zebrafish [85]. According to the study, Re increased ALP activity in MC3T3-E1 cells in a dose-dependent manner. ALP activity was stimulated up to 200% and 350% by re-treatment at concentrations of 50 and 100 μM for 14 days. The zebrafish scales exposed to ginsenoside Re (50 μM) for 35 days exhibited elevated calcium levels, which were analyzed by the Alizarin Red S staining. The strength of Alizarin Red S staining was 2.4 times greater than that observed in the untreated control zebrafish scales. In another study, the same research group reported that ginsenoside Re inhibited osteoclast differentiation in macrophages from mouse bone marrow and zebrafish [86]. Re-treatment significantly decreased TRAP activity in macrophages from mouse bone marrow in a dose-dependent manner. At concentrations of 5 μM, ginsenoside Re decreased TRAP activity by approximately 33% compared to the untreated control group. Re-treatment (5 μM) for 35 days resulted in a 63% decrease in TRAP intensity compared to the control zebrafish scale. Western blotting analysis showed that re-treatment induced the downregulation of NFATc1, c-Fos, and pErk and suppression of NF-κB pathway in macrophage from mouse bone marrow.

Gu et al. reported that ginsenoside Rg1 induced osteogenic differentiation of mesenchymal stem cells from rat bone marrow and accelerated the healing of rat tibial fractures [87]. The ALP activity in Rg1 (1 μM) treated mesenchymal stem cells was about 2-fold higher compared to the control cells. Micro-CT analysis revealed that BMD was significantly increased in the Rg1 treatment group in time time-dependent manner. Unfortunately, they did not perform a quantitative analysis of the micro-CT results. Mechanistically, Rg1 stimulated the BMP-2/Smad signaling pathway by regulating the translocation of the glucocorticoid receptor (GR) receptor into the nucleus. Chen et al. studied the anti-osteoporosis activity of Rg1 in osteoprogenitor cells from rat femur and high glucose-induced diabetic osteoporosis (Goto-Kakizaki) rat model [88]. The analysis of ALP and Alizarin Red S staining assay showed that Rg1 treatment (164.8 μM) for 7 days induced the differentiation of the isolated osteoprogenitor cells from rat femur into osteoblast. Although the treatment dose seemed relatively higher than those used in other studies, Rg1 increased the strength of ALP and Alizarin Red S staining up to 300 and 500-fold compared to the control, respectively. Quantitative micro-CT analysis also showed that Rg1 (10 mg/kg) administration for 12 weeks increased BMD by about 1.5-fold in diabetic osteoporosis (Goto-Kakizaki) rats compared with the control group. Authors suggested that Rg1 could facilitate the coupling between bone formation and blood vessel growth by stimulating osteoprogenitor cells exposed to high glucose conditions to release vascular endothelial growth factor (VEGF), thereby enhancing angiogenesis. Jiang et al. reported that Rg1 enhanced osteogenesis in MC3T3-E1 and DEX-induced osteoporosis zebrafish by modulating the G protein-coupled estrogen receptor (GPER)/PI3K/AKT pathway [89]. When compared to the DEX-treated MC3TC-E1 cells, the Rg1-treated MC3TC-E1 cells (50 μM, 7 days) displayed an approximately 3-fold increase in ALP activity, which was a similar effect of E2 treatment (100 nM). The number of Alizarin Red S staining positive vertebrae of DEX-induced osteoporosis zebrafish was increased by about 2.5-fold with 50 µM of Rg1 treatment for 10 days. Authors proposed that Rg1 might regulate osteogenesis by mediating GPER expression within the PI3K/AKT signaling pathway in glucocorticoid-induced osteoporosis.

Ginsenoside Rg3 inhibited osteoclast differentiation in RAW264.7 cells via suppression of NF-κB and MAPK signaling pathways [90]. Rg3 (10 μM) treatment for 7 days reduced RANKL-induced activation of TRAP activity up to about 20% in RAW264.7 cells. Authors indicated that Rg3 inhibited the RANKL-induced activation of NF-κB and MAPK signaling pathways in RAW264.7 cells. Also, they showed that Rg3 modulated cathepsin K, an important enzyme in bone resorption signaling cascades by in silico molecular docking interaction study. The study combined in silico and in vitro approaches to elucidate the mechanisms of the working mechanism of Rg3. Zhang et al. reported that Rg3 effectively mitigated glucocorticoid-induced osteoporosis through modulation of the bone morphogenic protein-2 (BMP-2)/BMP receptor 1A (BMPR1A)/Runx2 signaling pathway [91]. In primary osteoblasts from rat skulls, DEX substantially reduced ALP activity, while treatment of Rg3 (10 µM) for 14 days notably enhanced ALP activity by about 2-fold compared to the DEX-treated cells. Interestingly, the author showed that high doses (20 and 100 µM) of Rg3 caused osteoblasts cell death. Using the DEX-induced osteoporosis rat model, the author demonstrated that Rg3 (20 mg/kg) administration for 5 weeks recovered DEX-induced BMD reduction by about 1.4-fold compared to the DEX-treated rat group. Song et al. reported that Rg3 had a protective effect against AlCl3-induced osteoporosis in rats by regulation of oxidative stress [92]. X-ray image analysis showed the BMD (about 210 mg/cm2) of femoral metaphysis was higher in the Rg3 administrated rat group (20 mg/kg for 1 month) than that (about 180 mg/cm2) of the AlCl3 group. Rg3 reduced malondialdehyde and reactive oxygen species (ROS) levels but increased glutathione peroxidase and superoxide dismutase activity of femora in the Rg3 administrated rat group compared to the AlCl3 group. Zhang et al. explored the anti-osteoporosis effect of Rg3 in MC3T3-E1 cells and ovariectomy-induced osteoporosis rats via promoting autophagy and the AMPK/mTOR signaling pathway [93]. Rg3 treatment (20 μM) for 5 days was found to increase ALP activity in MC3T3-E1 cells by about 3-fold compared to the control. These effects were reversed by Compound C (an AMPK inhibitor). Rg3 administration (20 mg/kg) for 5 weeks recovered BMD declines by 2-fold in ovariectomy-induced osteoporosis rats. Song et al. demonstrated that Rg3 attenuated AlCl3-induced bone impairment both in vivo and in vitro by modulating the TGF-β1/Smad signaling pathway [94]. Rg3 treatment (10 μM) for 24 h resulted in a significant increase (about 50%) in the ALP activity of MC3T3-E1 cells compared to AlCl3-treated cells. The administration of Rg3 (20 mg/mL) for 1 month reduced the cell apoptosis and histologically impaired modifications caused by AlCl3 in the femurs of rats. Unfortunately, authors did not provide quantitative analysis such as measurement of BMD by CT to monitor bone healing progress.

Siddiqi et al. reported the anti-osteoporotic properties of the mixture of ginsenosides Rg5 and Rk1 in MC3T3-E1 cells [95]. The mechanism of action of the Rg5 and Rk1 mixture appeared to involve the activation of BMP-2 and Runx2 pathways. The treatment of Rg5 and Rk1 mixture (20 μg/mL) increased ALP activity (1.8-fold) and Alizarin Red S staining density (2.5-fold) in MC3T3-E1 cells for 12 days. The same research group reported that Ginsenoside Rh1 promoted osteoblast differentiation in MC3T3-E1 cells [96]. Similar to the mechanism of action of the Rg5 and Rk1 mixture, the effects of Rh1 were mediated through the BMP-2 and Runx2 signaling pathways. Rh1 treatment (10 μM) for 12 days increased ALP activity by 1.5-fold in MC3T3-E1 cells.

Ginsenoside compound K (CK) could be a potential therapeutic agent for treating fractures by promoting osteogenesis [97]. Alizarin Red S staining analysis showed that CK treatment (10 μM) for 14 days increased the formation of calcium deposits in bone marrow mesenchymal stem cells from rats. In the rat femoral fracture model, micro-CT results showed that CK administration (500 μM) for 4 weeks increased BMD averagely by 1.5-fold. The mechanism of action involved the activation of β-catenin and Runx2 in fracture calluses. Interestingly, authors suggested that CK increased angiogenesis around the fracture callus by the formation of H-type vessels. In another study, CK attenuated RANKL-mediated osteoclast differentiation in RAW264.7 cells and OVX-induced osteoporosis in a mouse model [98]. TRAP staining analysis showed that CK treatment (10 μM) for 5 days reduced the number of multinucleated RAW264.7 cells by about 50% compared to the RANKL-treated control cells. Authors indicated that CK suppressed the phosphorylation of NF-κB p65 responsible for the activation of RAW264.7 cells. In vivo experiments confirmed that CK administration (10 mg/mL) for 8 weeks increased OVX-induced BMD loss in rats by about 2-fold compared to the OVX group through alleviating oxidative stress in bone tissue.

### 3.2. Extracts

Recent research findings have demonstrated that not only ginsenosides but also ginseng extract show potential in treating osteoporosis. In fact, recent studies highlight the multifaceted approach of ginseng extract in protecting bone health, addressing both the enhancement of bone formation and the suppression of bone resorption in various experimental models.

Kim et al. investigated the potential of Korean Red Ginseng extract as a natural therapeutic agent to prevent or treat DEX-induced osteoporosis both in vitro and in vivo [99]. MC3T3-E1 cells exposed to DEX exhibited reduced ALP activity. However, when cells were treated with both DEX and Korean Red Ginseng extract (125 μg/mL) for 7 days, ALP activity was increased by 1.8-fold compared to the DEX-treated cells. Interestingly, the treatment of a higher dose of Korean Red Ginseng extract (250 and 500 μg/mL) decreased the ALP activity compared to the Korean Red Ginseng extract (125 μg/mL) treated cells. Micro-CT image analysis showed that DEX mice exhibited a decrease in trabecular BMD within the femur. In contrast, mice administrated with Korean Red Ginseng treatment (500 mg/mL) for 5 weeks demonstrated a notably increased BMD. The authors did not provide the present quantitative analysis results of the micro-CT image. Lee et al. reported that ginseng extract and ginsenoside Re could be promising natural agents for preventing osteoporosis, especially in cases of estrogen deficiency [100]. The treatment of ginseng extract (50 μg/mL) for 5 days inhibited RANKL-induced TRAP activity by 30% in RAW264.7 cells compared to the RANKL-treated cells, which seemed to have a similar effect of E2 (10 nM). HPLC analysis showed that the ginseng extract contained various ginsenosides. Among them, ginsenoside Re was shown to be a major component of the extract to suppress osteoclast differentiation in RAW264.7 cells. In vivo studies using OVX-induced osteoporosis rat models demonstrated administration of ginseng extract (300 mg/kg) for 8 weeks increased BMD by about 25% compared to the OVX rats. Another study demonstrated that ginseng leaf extract attenuated RNAKL-induced osteoclast differentiation in vitro by downregulation of high mobility group box-1 (HMGB1) and upregulation of (nuclear factor-erythroid 2-related factor 2) Nrf2 and heme oxygenase-1 (HO-1) [101]. Although the authors did not provide the quantitative results, TRAP staining analysis showed that the treatment of ginseng leaf extract (1 mg/mL) for 5 days with RANKL decreased TRAP-positive RAW264.7 cell number as much as the vehicle control. Seo et al. discussed the effects of ginseng-derived extracellular nanovesicles on osteoclast differentiation [102]. Ginseng-derived extracellular nanovesicles were isolated using sucrose gradient ultracentrifugation and showed about 71.42 nm of diameter. Ginseng-derived extracellular nanovesicles contained high levels of ginsenoside Rb1 and Rg1. The treatment of ginseng-derived extracellular nanovesicles (1 μg/mL) for 4 days exhibited about 20% fewer TRAP-positive macrophages from mouse bone marrow than the RANKL control group. In vivo, studies using lipopolysaccharide-induced osteoporosis mouse models demonstrated ginseng-derived extracellular nanovesicles administration (1 mg/mL, 7 days) recovered almost the reduced BMD in lipopolysaccharide-induced osteoporosis mouse model. Mechanistically, ginseng-derived extracellular nanovesicles caused attenuation of the expression level of pIκBα, pJNK, pp38, and pERK activated by RANKL in mouse bone marrow-derived macrophage.

### 3.3. Summary

As shown in Table 2, recent studies reveal that various ginsenosides and ginseng extracts were explored to have anti-osteoporosis activity by regulating signaling pathways involved in activating osteoblast or inhibiting osteoclast using several kinds of in vitro and in vivo models. Similar to the research results on the anti-osteosarcoma activity of phytochemicals from ginseng, there was no report about the anti-osteoporosis activity of alkaloids or peptides from ginseng based on the search results from 2014 to 2024. There were five publications on the anti-osteoporosis activity of Rg3. Rg3 is the most extensively studied compound in the field of osteoporosis, which makes it a compelling subject for continued investigation in the field of bone health and osteoporosis treatment. For example, Siddiqi et al. reported that fermented ginseng extraction to enrich Rg3 displayed pro-osteoblastic activity in MC3T3-E1 cells [103]. Fermented ginseng extraction treatment increased ALP activity and the expression of type-1 collagen. The authors showed that after fermentation, the levels of Rh1 (11%) and Rg2 (16%) slightly increased, while Rg3 (33%) significantly increased. The in vitro effects of ginsenosides may not directly correlate with their in vivo efficacy in treating osteoporosis. For instance, Rb1 showed promise in promoting MSC differentiation into osteoblasts in vitro but it did not translate to significant bone preservation in the OVX rat model [79]. There is an interesting study about the identification of novel targets involved in treating osteoporosis by ginsenoside CK using a network pharmacology approach [104]. Authors showed that CK interacted with c-Fms protein, stabilized by hydrogen bonds and hydrophobic interactions. Authors suggested that CK could interfere with osteoporosis progression through the c-Fms-mediated MAPK and PI3K signaling axis that regulates osteoclast differentiation.

In vitro assay model, the most effective dose of ginsenosides ranged from 1 to 200 µM, which was evaluated by either ALP or TRAP assays. ALP and TRAP assays are two different kinds of techniques used in bone research, each serving a distinct purpose. ALP targets osteoblast activity (bone formation), while TRAP targets osteoclast activity (bone resorption) [105]. In the case of ginseng extract, the most effective dose was from 50 μg/mL to 1 mg/mL. The treatment time with ginsenosides varied among studies and most experiments were performed for 7 or 14 days. Both ALP and TRAP assays were used to assess the rate of differentiation to osteoblast or osteoclast cells and it requires several days to weeks to accumulate sufficient levels of enzymatic products necessary to induce cell differentiation. Many studies adapted MC3T3-E1 osteoblast or RAW264.7 osteoclast cells as an in vitro model system as shown in Table 2. The main target signaling pathways of ginsenosides and ginseng extract involved Wnt/β-catenin, TGF-β/Smad, BMP-2/Smad, BMP-2/Runx2, PI3K/Akt for induction of osteoblasts differentiation and NF-κB, STAT3, and MAPK for suppression of osteoclast differentiation. Also, ginsenosides and ginseng extract showed anti-osteoporosis activities through regulation of osteoblast or osteoclast and suppression of oxidative stress. There were several kinds of in vivo models such as glucocorticoid-induced osteoporosis animals (mice, rats, and zebrafish), ovariectomized osteoporosis animals (mice and rats), AlCl3-induced osteoporosis rats, lipopolysaccharide-induced osteoporosis mice, ketogenic-diet-induced osteoporosis mice, tibial fractured rats, and femoral fractured rats. After inducing osteoporosis primarily through compounds or physical damage, the anti-osteoporotic effects were examined by orally administering specific ginsenosides or ginseng extracts and applying various osteoporosis recovery methods. Although it varied among studies, researchers primarily administered ginsenosides at a dose of 20 mg/kg for up to 12 weeks. In most vivo studies, BMD (Bone Mineral Density) was presented and analyzed as the most fundamental anti-osteoporosis indicator. Additionally, some studies also showed results using other indicators such as BV/TV (Bone Volume/Total Volume), Tb.N (Trabecular Number), Tb.Sp (Trabecular Separation), and SMI (Structure Model Index). While these findings are promising, it is still important to note that most of the research has been conducted in vitro on cell lines and in vivo models. Further studies, especially clinical trials, are needed to fully establish the efficacy and safety of ginsenosides or ginseng extract as a treatment for osteoporosis in humans.

## 4. Phytochemicals from Ginseng in Management of Osteoarthritis

### 4.1. Ginsenosides

Phytochemicals from ginseng have demonstrated significant effects on rheumatic diseases including osteoarthritis, offering promising potential as therapeutic agents [75,106,107]. Especially, recent studies discussed how ginsenoside Rb1, Rg1, Rg3, Rg5, Ro, and CK reduced inflammation and exhibited potential as therapeutic candidates for osteoarthritis management.

Ginsenoside Rb1 was demonstrated to show potential anti-inflammatory effects in osteoarthritis treatment [108]. According to the study, Rb1 treatment (80 μM) for 24 h decreased IL-1β-induced NO production by 46% compared to the IL-1β treated SW1353 cells, a kind of human chondrosarcoma cell lines. Rb1 inhibited the protein expression of inducible nitric oxide synthase (iNOS), a key enzyme in NO production. Rb1 suppressed NF-κB activation through increased IκBα protein content, reduced nuclear translocation of p65 protein, and decreased DNA binding activity of NF-κB. In another study, Rb1 showed promising effects in inhibiting MMP-13 expression and promoting type II collagen expression through regulation of the Notch signaling pathway in osteoarthritis [109]. Treatment of SW1353 cells with Rb1 (80 µM) for 24 h reduced IL-1β-induced protein expression of MMP-13 up to about 70% compared to the cells only treated with IL-1β. In the anterior cruciate ligament transection (ACLT) osteoarthritis rat model, Rb1 treatment (80 μM) for 6 weeks ameliorated cartilage degradation and improved histological scores by about 50%. Similarly, Chen et al. reported that Rb1 treatment reduced IL-1β levels, attenuated cartilage degeneration, and lowered histologic damage scores by suppressing MMP-13 and type X collagen expression in C5.18 cells (rat chondrocyte) and rat osteoarthritis model combining ACLT and medial cruciate ligament transection (MCLT) [110]. Aravinthan et al. reported that Rb1 displayed anti-inflammatory and chondro-protective effects in monoiodoacetate (MIA)-induced and ovariectomized osteoarthritis rat model [111]. X-ray radiography analysis revealed that Rb1 (10 μg/kg) administration for 4 weeks decreased knee joint thickness by about 70% compared to the control osteoarthritis rat groups. The authors showed that the mRNA expression of MMP-13 in chondrocytes from the Rb1 (10 μg/kg) administration group was about half of the control osteoarthritis group. Luan et al. reported the therapeutic potential of Rb1 in attenuating MIA-induced osteoarthritis by modulating the miR-21-5p/FGF18 axis [112]. Rb1 (10 μM) treatment for 24 h reduced miR-21-5p–mediated inhibition of FGF18, which was associated with activation of matrix synthesis and cell proliferation in chondrocytes from healthy rats. Similarly, in MIA-induced osteoarthritis rat models, Rb1 (10 mg/kg) administration for 2 weeks improved joint histology, suppressed pro-inflammatory cytokines (e.g., IL-1β and IL-6), and enhanced chondrocyte viability. Mohammad et al. reported a protective effect of Rb1 against osteoarthritis pathogenesis-induced hollow trephine on the femur trochlea of rabbits by down-regulation of p-Akt, p-P38, and p-P65, which were associated with inflammatory processes and cartilage degradation in osteoarthritis [113]. The study utilized a rabbit knee osteoarthritis model, where hydrogel-based Rb1 sheets were inserted into the knee joint. Hydrogel-based Rb1 (100 μg/kg) administration exhibited dose-dependent chondroprotective effects, as evidenced by macroscopic and micro-CT investigations.

Another ginsenoside Rg1 was reported to show protective effects against IL-1β-induced chondrocyte apoptosis by modulating the PI3K/Akt signaling pathway [114]. Rg1 treatment (10 μg/mL) for 72 h increased the cell viability of chondrocytes from healthy rats by about two-fold compared to the IL-1β treated group. Rg1 suppressed chondrocyte apoptosis by upregulation of pAkt, Bcl2, and TIMP-1 and downregulation of Bax and MMP-13. Wendan et al. reported that Rg1 prevented osteoarthritis pathogenesis by targeting inflammatory, apoptotic, and ECM-degrading pathways in human chondrocytes and rat ACLT models [115]. Rg1 (10 μg/mL) treatment for 24 h suppressed IL-1β-induced mRNA expression of MMP-13 and COX-2 by 7.5- and 2.2-fold, respectively, in human chondrocytes from osteoarthritis patients with knee replacement. Oral administration of Rg1 (60 mg/kg for 12 weeks) reduced cartilage erosion, collagen II loss, and MMP-13 levels. Zhiqiang et al. reported that Rg1 exerted protective effects against IL-1β-induced apoptosis in primary human chondrocytes [116]. Rg1 (100 μg/mL) treatment for 72 h restored the proliferation of primary human chondrocytes inhibited by IL-1β by about 9-fold. Rg1 treatment reduced ROS and MDA production. Rg1 treatment also downregulated Bax expression but upregulated Bcl2 expression. Huaisen et al. reported the therapeutic potential of Rg1 in countering thiram-induced osteoarthritis in chickens by modulating apoptosis and angiogenesis [117]. The cell viability of primary chicken chondrocytes was decreased to 75% by thiram exposure. Rg1 treatment (40 μM) for 24 h recovered the cell viability by about 95% compared to the control group without thiram and Rg1. Rg1 administration (40 mg/kg) for 18 days reduced tibial dyschondroplasia severity, improved growth plate structure, and restored cartilage matrix integrity in thiram-induced osteoarthritis chickens. Yanwei et al. reported that the combined therapy of Rg1 and adipose-derived stem cells embedded in a hyaluronic acid matrix displayed the anti-osteoarthritic efficacy in papain-induced osteoarthritis rabbits model [118]. Rg1 treatment (40 μM) for 5 days increased the proliferation by about 10% in chondrocytes differentiated from healthy rabbit adipose stem cells. The administration 1:1 (V:V) mixture of hyaluronic acid and adipose stem cells (2 × 10^7^/mL) suspension in 40 μM of Rg1 for 9 days reduced papain-induced osteoarthritis severity of rabbit model, which was evidenced by histological (scanning electron microscopy) improvements in cartilage thickness. Rg1 upregulated the expression of type II collagen and TIMP-1 but downregulated the expression of MMP-13.

Ginsenosides Rg3 treatment (20 μM) for 24 h reduced MMP-13 expression in IL-1β-stimulated SW1353 human chondrocytes by 58% compared to the IL-1β treated control [119]. In addition, Rg3 (2 μM) treatment for 3 days inhibited glycosaminoglycan release in IL-1β-stimulated rabbit cartilage cultures by 12.6% compared to the IL-1β treated control. In another study, Ching-Hou et al. reported a more detailed working mechanism of Rg3 in chondrocyte protection [120]. In TNF-α-stimulated human chondrocyte TC28a2, Rg3 (20 μM) treatment for 24 h upregulated SIRT1 expression by about 2-fold, which activates the SIRT1/PGC-1α/SIRT3 signaling axis. This pathway mitigated mitochondrial dysfunction by reducing ROS accumulation. The resultant decrease in ROS attenuated caspase-dependent apoptosis and suppresses pro-inflammatory mediators such as IL-8 and MMP-9. Additionally, Rg3 inhibited TNF-α-induced NF-κB nuclear translocation by down-regulating p38 MAPK phosphorylation.

Ginsenoside Rg5 showed the chondroprotective effects in chondrocytes from healthy rats and the MCLT osteoarthritis rat model by inhibiting chondrocyte apoptosis and preserving cartilage matrix integrity through modulation of inflammatory pathways [121]. Rg5 (15 μM) treatment for 48 h reduced apoptosis of chondrocytes from healthy rats by 98% compared to the IL-1β treated control cells. Rg5 (15 mg/kg) administration for 1 month decreased apoptosis of chondrocytes in MCLT osteoarthritis rats by 98% compared to control rats, leading to prevention of synovial disintegration and preserving joint functionality.

Ginsenoside CK was demonstrated to exert cartilage-protective effects in chondrocytes from osteoarthritis patients through the regulation of IL-1β-induced MAPK pathways [122]. CK (5 μM) treatment for 24 h suppressed IL-1β-driven NO production by 95.8% compared to IL-1β-treated control chondrocytes. CK treatment reduced IL-1β-induced iNOS, MMP-13, pJNK, pp38, and pErk expression and inhibited glycosaminoglycan release in chondrocytes from osteoarthritis patients. Yuguo et al. reported the therapeutic potential of CK in osteoarthritis by targeting NLRP3 inflammasome-mediated pyroptosis [123]. CCK8 assay using primary mouse chondrocytes revealed that TNF-α reduced cell viability by up to 50% compared to the control cell but CK (5 μM) treatment for 48 h restored cell viability by about 75%. Mechanistically, CK suppressed NLRP3 inflammasome activation by reducing TNF-α-induced upregulation of NLRP3. In the MCLT osteoarthritis mouse model, CK administration (40 mg/kg) for 8 weeks decreased Osteoarthritis Research Society International (OARSI) scores from 20 to 15. CK also reduced cartilage degradation and normalized collagen II/MMP13 expression ratios in MCLT osteoarthritis mouse. Similarly with this study, Yicheng et al. reported that CK exhibited anti-osteoarthritis efficacy by regulating pyroptosis through targeting endoplasmic reticulum stress-mediated activation of the IRE1α-TXNIP-NLRP3 axis [124]. In IL-1β-stimulated chondrocytes, CK (30 nM) treatment for 24 h reduced the protein expression of MMP-13 by about 50%. In monoiodoacetate-induced rat osteoarthritis models, CK administration (80 mg/kg) for 7 weeks decreased the OARSI and Mankin scores by about 45%. CK inhibited IRE1α phosphorylation, reducing thioredoxin-interacting protein (TXNIP) translocation to NLRP3 and subsequent inflammasome assembly. This suppression was reversed by the endoplasmic reticulum stress inducer tunicamycin, confirming the dependency of CK on endoplasmic reticulum stress modulation.

### 4.2. Summary

As shown in Table 3, recent studies demonstrated the anti-osteoarthritic activity of various kinds of ginsenosides through modulation of inflammatory pathways and cartilage protection in both in vitro and in vivo models. There was no report about the anti-osteoarthritic activity of alkaloids or peptides from ginseng based on the search results from 2014 to 2024. During this period, researchers reported 17 publications. Rb1 and Rg3 are the most extensively studied ginsenosides in the field of osteoporosis. There were six and five publications on the anti-osteoporosis activity of Rb1 and Rg1, respectively. Ginsenosides Rb1 and Rg3 are structurally related ginsenosides derived from ginseng but they exhibit distinct features in their glycosylation patterns. Both share a dammarane-type tetracyclic triterpenoid backbone (protopanaxadiol). Both have two glucose units attached at C3 via a β-(1→2) glycosidic bond. Rb1 possesses two glucose units linked via β-(1→6) but Rg3 is boned with a free hydroxyl group at C20 instead of glucose. Rb1 and Rg3 are the most thoroughly researched ginsenoside in the field of osteoarthritis, making it an especially promising candidate for ongoing studies related to the treatment of osteoarthritis. For example, Tingting et al. reported that a novel biodegradable silk fibroin-gelatin porous scaffold co-loaded with ginsenoside Rb1 and TGF-β1 was developed to address inflammation and promote cartilage regeneration [125]. The scaffold had a porous microstructure designed for suitable mechanical strength, controlled degradation, and sustained release of both Rb1 and TGF-β1. Rat bone marrow-derived mesenchymal stem cells seeded onto the scaffold exhibited enhanced proliferation and chondrogenic differentiation compared to non-loaded controls. The scaffold significantly suppressed inflammatory gene expression and reduced inflammation in C5.18, rat chondrocyte cell lines under IL-1β stimulation. In a rat osteochondral defect model, the implantation of the scaffold led to effective regeneration of hyaline cartilage after 12 weeks compared to other groups. In another study, Yongsheng et al. demonstrated that Rg1 incorporated poly 3-hydroxybutyrate-co-3-hydroxyvalerate (PHBV) fibrous scaffolds mitigated IL-1β-induced dysregulation in rabbit articular chondrocytes [126]. This engineered scaffold maintained chondrocyte morphology and phenotypic stability under IL-1β stimulation conditions compared to the unmodified PHBV. The scaffold also displayed chondrocyte protective effects by down-regulating the expression of MMP-1 and upregulating expression of SOX9, type II collagen, and aggrecan. In research similar to the two studies above, Jun-Ho et al. explored the development of CK-loaded adhesive patches for cartilage tissue regeneration [127]. Authors functionalized hydrocaffeic acid-conjugated chitosan patches with CK, demonstrating stable adhesion to cartilage defects and sustained CK release. These patches inhibited apoptosis of chondrocytes, MMP-13 activity, and NF-κB signaling pathway in osteoarthritis mice model induced by destabilization of the medial meniscus surgery.

As shown in Table 3, a variety of in vitro and in vivo models were used to study the anti-osteoarthritis activity of ginsenosides, which was chosen based on research goals and disease mechanisms. In vitro models, the most effective dose of each ginsenoside ranged from 2 to 125 μM and was tested by various kinds of elementary assay. These assays included quantification of nitric oxide (NO), Western blot analysis of MMP-13, RT-PCR analysis of MMP-13, CCK8 assay, MTT assay, and TUNEL assay in chondrocyte cell line or primary chondrocyte extracted from knee tissue of human or animal. The chondrocyte cells were treated by each ginsenoside for 24 h in the most in vitro assays. In vivo models can be grouped by animal species and by the method of osteoarthritis induction. Common animal species included small animals such as mice and rats. These animals were widely used for genetic, molecular, and mechanistic studies since they allow for genetic modification and are cost-effective. Researchers used chemically induced animal models by injection of chemicals such as papain and monosodium iodoacetate into the joint to induce osteoarthritis. In addition, surgically induced models were adapted through procedures such as anterior cruciate ligament transection, medial cruciate ligament transection, or destabilization of the medial meniscus.

Chondrocyte viability is a critical focus in anti-osteoarthritis therapy development because these cells are the sole producers and maintainers of cartilage extracellular matrix in articular cartilage [128,129,130]. In osteoarthritis, excessive apoptosis of chondrocytes disrupts this balance, leading to extracellular matrix breakdown and irreversible cartilage loss. Thus, an assay system targeting chondrocyte viability and apoptosis could address the core pathological mechanism of osteoarthritis by preserving cartilage integrity. MMP-13, also known as collagenase-3, is a primary therapeutic target for osteoarthritis due to its critical role in cartilage degradation and disease progression [131,132,133]. MMP-13 cleaves type II collagen, the main structural component of articular cartilage, faster than other collagenases. Except for type II collagen, MMP-13 is known to degrade other matrix components such as proteoglycans and osteonectin, exacerbating joint damage. Type II collagen is the primary structural protein in cartilage, constituting 85–90% of articular cartilage collagen and 50% of all cartilage protein. Type II collagen plays an important role in cartilage structure and modulates osteoarthritis progression through immune tolerance and cartilage protection [134,135,136]. For example, James et al. reported that undenatured type II collagen supplementation alleviated knee osteoarthritis symptoms across multiple randomized controlled trials [137]. Targeting inflammation in the development of anti-osteoarthritis therapies is recognized as a rational and promising strategy [138,139,140]. In fact, recently reported ginsenosides showed anti-osteoarthritis activities through regulation of inflammation which drives the production of enzymes such as MMPs to break down the extracellular matrix of cartilage via regulation of pro-inflammatory cytokines such as IL-1β and TNF-α. Two clinical studies have investigated the anti-osteoarthritis effect of ginseng. Hye In et al. reported that red ginseng supplementation improved pain scores and anti-oxidative activity in postmenopausal women with hand osteoarthritis [141]. After 12 weeks, the red ginseng supplementation (3 g/day) group showed significant improvement in pain scores and DASH scores compared to the placebo group. Anti-oxidant enzyme (superoxide dismutase) activity increased and oxidative stress marker (malondialdehyde) levels decreased in the red ginseng supplementation group compared to the placebo group. Su-jin et al. reported that the group receiving *Panax ginseng* extracts showed significant enhancements in WOMAC osteoarthritis index scores [142]. Among women with osteopenia, daily supplementation with 3 g of *Panax ginseng* extracts for 12 weeks led to marked improvements in knee arthritis symptoms such as knee pains and stiffness of joints.

## 5. Current Limitations and Future Direction

Ginseng and its bioactive compounds show therapeutic potential in bone-related diseases. However, there are several limitations to using them as therapeutic agents or functional food ingredients to treat or prevent bone-related diseases. First, the specific composition and concentration of phytochemicals from ginseng can vary depending on the ginseng species, plant part used, and processing methods [143,144,145]. This variability in ginsenoside content can lead to inconsistent therapeutic effects. Thus, advanced techniques such as two-dimensional liquid chromatography and hybrid mass spectrometry are required to identify and quantify ginsenoside metabolites. Also, a proper extract method needs to be considered for specific ginsenoside production. Secondly, it is hard to extract large quantities of phytochemicals from ginseng for industrial production [146,147]. It could be one of the options to consider biotransformation using synthetic biology tools [148]. For example, protopanaxatriol can also be produced by enzymatic conversion of other ginsenosides or precursors in engineered microorganisms [149]. In addition, investigating the synergistic effects of ginsenosides with other phytochemicals or conventional drugs could lead to more effective therapeutic strategies [150]. Thirdly, the safety and side effects of phytochemicals from ginseng should be carefully considered for dosage, duration, and individual susceptibility [151,152]. Potential concerns include gastrointestinal disturbances and the risk of immune suppression, particularly in patients receiving concurrent chemotherapy for osteosarcoma [153]. For example, some studies have raised alarms about the possibility of ginsenosides having tumor-promoting effects under certain conditions, highlighting the need for further investigation to clarify their role in cancer treatment and to establish appropriate clinical guidelines [154]. Thus, continued exploration of targeted delivery systems and combination therapies could significantly improve the treatment efficacy of phytochemicals from ginseng addressing safety concerns associated with conventional approaches. Fourthly, the poor bioavailability of phytochemicals from ginseng due to their hydrophilicity hampers their absorption in the gastrointestinal tract [155]. The degradation of ginsenosides by intestinal flora can affect their pharmacokinetics and pharmacological efficacy, necessitating studies on their metabolic pathways [156]. In humans, the half-lives of ginsenoside are usually less than 24 h, limiting their duration of action [157]. Some ginsenosides can be degraded or transformed during processing, which may affect their intended therapeutic effects. To solve the lower bioavailability of phytochemicals from ginseng, it is necessary to design the appropriate dosing regimens and sustained administration approach to maintain effective plasma concentrations. Fifthly, in-depth studies on the specific molecular mechanisms of phytochemicals from ginseng are crucial for understanding their therapeutic potential in bone-related disease to avoid unintended effects and optimize targeted therapy [75,158]. For example, ginsenosides modulate diverse pathways such as NF-κB, Wnt/β-catenin, and Nrf2, which complicates targeted therapy and increases the risk of unintended effects in hormone-sensitive conditions [159,160,161]. To minimize unintended effects, personalized approaches such as pathway-specific delivery systems and biomarker-guided dosing, could mitigate risks while preserving the benefits of phytochemicals from ginseng. Sixthly, several other plants contain other phytochemicals that can offer similar health benefits and biological activities, making them valuable alternatives or complements in therapeutic and nutritional contexts [162,163]. *Astragalus membranaceus*, commonly used in traditional Chinese medicine, contains triterpenoid saponins known as astragalosides. These compounds have been shown to have immunomodulatory, anti-inflammatory, and anti-oxidant effects, similar to some of the biological activities of ginsenosides [164,165]. *Eleutherococcus senticosus* (Siberian ginseng) contains eleutherosides, a class of compounds structurally different from ginsenosides but with analogous adaptogenic, immunomodulatory, and anti-fatigue properties. It is widely used as a ginseng alternative in traditional medicine [166]. Seventhly, more clinical trials with high quality are essential to validate the efficacy and safety of phytochemicals from ginseng in treating bone-related diseases. For this issue, several critical factors must be addressed based on existing research gaps and methodological insights. For example, variability in ginseng composition requires rigorous chemical characterization to ensure consistency across trials [167]. Long-term studies are needed to assess sustained benefits, since bone remodeling is a slow process [168]. High-risk groups such as elderly or postmenopausal individuals are carefully selected to evaluate efficacy in preventing osteoporosis progression [142]. Thus, clinical studies should also address issues of variability, bioavailability, and effectiveness in humans in reference to most current evidence derived from in vitro and in vivo results. Eighthly, most research on bone health benefits of ginseng was performed by Asian countries such as China, Korea, and Japan. Clinical trials in multiple countries could assess efficacy across genetic and lifestyle backgrounds. Although there seems no reference to clinical trials on ginseng for bone-related diseases, emerging studies are paving the way for broader global adoption to expand the health benefits of ginseng [169,170,171]. For instance, a comprehensive review summarized evidence from 19 meta-analyses, confirming that ginseng intake is associated with improvements in fatigue, physical function, sexual function, menopausal symptoms, metabolic indicators, inflammatory markers, and cardiovascular health. These ongoing studies could facilitate broader acceptance of the health benefits of ginseng in a global population. These limitations highlight the need for further research to improve the bioavailability, stability, and clinical meaning of phytochemicals from ginseng for their effective use as therapeutic agents or functional food ingredients. By addressing these limitations and pursuing these future directions, researchers can unlock the full therapeutic potential of ginseng-derived phytochemicals in treating bone-related diseases.

## 6. Conclusions

Ginseng has been utilized in traditional medicine for centuries, celebrated for its diverse health benefits. Recent studies suggest that phytochemicals from ginseng, particularly ginsenosides, offer new avenues for treating bone-related diseases such as osteosarcoma, osteoporosis, and osteoarthritis. These phytochemicals show promise in modulating critical signaling pathways related to specific bone-related diseases in vitro and in vivo models. Limitations of phytochemicals from ginseng included variability, bioavailability, and insufficient clinical data highlighting the need for standardized formulations, rigorous clinical validation, and advanced delivery systems to optimize its role in bone health therapeutics. Overall, the ongoing research into the applications of ginseng-derived phytochemicals presents exciting possibilities for enhancing the management of osteosarcoma, osteoporosis, and osteoarthritis.

## Figures and Tables

**Table 1:** Phytochemicals from ginseng with anti-osteosarcoma activity.

No.	Phytochemicals	Cell Model	Viability Assay	Dose ^a^, Time	Animal Model	Dose, Time	Mechanism	Year, Ref.
**1**	Ginsenoside Rf	MG63	MTT	11 μM, 24 h	N/A	N/A	Induction of apoptosis	2014, [58]
**2**	Ginsenoside Rg3	MG63	MTT	150 μM, 24 h	N/A	N/A	Induction of double-strand DNA damage	2014, [22]
**3**	Ginsenoside Rg3	MG63	CCK8	2.5 μM, 24 h	N/A	N/A	Induction of apoptosis, inhibition of migration, suppression of PI3K/Akt/mTOR pathway	2018, [59]
**4**	Ginsenoside Rg3	MG63	EDU	80 μM, 24 h	Xenograft mouse (MG63)	20 mg/kg, 21 days	Induction of apoptosis, inhibition of migration and invasion, regulation of circ_0003074/miR-516b-5p/KPNA4 axis	2021, [60]
**5**	Ginsenoside Rg3	MG63	MTT	80 μM, 24 h	N/A	N/A	Inhibition of migration, suppression of Wnt/β-Catenin and EMT pathway	2020, [61]
**6**	Ginsenoside Rg5	MG63	MTT	0.64 μM, 24 h	N/A	N/A	Induction of apoptosis and autophagy, suppression of PI3K/Akt/mTORC1 pathway	2021, [62]
**7**	Ginsenoside Rh2	U2OS	MTT	8 μM, 48 h	N/A	N/A	Induction of apoptosis, inhibition of migration, suppression of PI3K/Akt/mTOR and NF-κB pathway, activation of MAPK pathway	2020, [64]
**8**	Ginsenoside Rh2	MG63	MTT	50 μM, 36 h	N/A	N/A	Induction of apoptosis, inhibition of migration and invasion, suppression of PI3K/Akt/mTOR and EMT pathway, activation of MAPK pathway	2021, [65]
**9**	Ginsenoside CK	MG63	MTT	20 μM, 72 h	N/A	N/A	Induction of apoptosis, inhibition of migration and invasion, suppression of PI3K/Akt/mTOR pathway	2020, [66]
**10**	Ginsenoside Rg1	MG63	CCK8	250 μM, 24 h with 6 μM Timosaponin AIII	N/A	N/A	Induction of apoptosis, inhibition of migration, suppression of MAPK pathway	2020, [67]
**11**	Ginsenoside Rb1, Rc	MG63	CCK8	250 μM, 24 h with 6 μM Timosaponin AIII	N/A	N/A	Induction of apoptosis, inhibition of migration, suppression of MAPK pathway	2019, [68]
**12**	Ginsenoside Rg3	143B	Colony formation	80 μg/mL, 7 days with 0.3125 μg/mL doxorubicin	Xenograft mouse (143B)	20 mg/kg, 21 days with 2 mg/kg doxorubicin	Inhibition of migration and invasion, suppression of Akt/mTOR, HIF1α/VEGF, and EMT pathway	2023, [69]
**13**	Ginsenoside CK	143B	MTT	10 µM, 72 h with 5 µM cisplatin	Xenograft mouse (143B)	10 mg/kg, 28 days with 10 mg/kg doxorubicin	Induction of apoptosis	2018, [70]
**14**	(20S)-Protopanaxatriol	MG63	MTT	0.5 μM, 24 h with 1 μM RSL3	Xenograft mouse (MG63)	20 mg/kg, 28 days with 20 mg/kg IKE	Increase in sensitivity to ferroptosis, increases in ACSL4 expression	2024, [71]
**15**	Polysaccharide	MG63	CCK8	20 μM, 24 h	N/A	N/A	Induction of apoptosis and autophagy, suppression of Ak and p38	2017, [73]

Dose ^a^: IC50 value for single treatment of ginsenoside, combinatorial effect dose for double treatment of ginsenoside and other compounds. MTT: 3-(4,5-dimethylthiazol-2-yl)-2,5-diphenyltetrazolium bromide. CCK8: Cell counting kit 8. EDU: 5-ethynyl-2′-deoxyuridine. N/A: Not available.

**Table 2:** Phytochemicals from ginseng with anti-osteoporosis activity.

No.	Phytochemicals	Cell Model	Assay ^a^	Dose ^b^, Time	Animal Model	Dose, Time	Mechanism	Year, Ref.
**1**	Ginsenoside Rb1	Osteoblasts from rat skull	CCK8	0.0145 mg/mL, 24 h	N/A	N/A	Increase in osteoblasts viability, upregulation of TGF-β1, BMP-2, IGF-I, and Cbfα1, inhibition of oxidative stress	2016, [77]
**2**	Ginsenoside Rb1	Osteoblasts from rat skull	ALP	0.0145 mg/mL, N/A	Glucocorticoid-induced osteoporosis rats	6 mg/kg, 12 weeks	Induction of osteoblasts differentiation, regulation of AHR/PRELP/NF-κB pathway	2022, [78]
**3**	Ginsenoside Rb1	Mesenchymal stem cells from rat bone marrow	ALP	10 μM, 7 days	Ovariectomized osteoporosis rats	6 mg/kg, 12 weeks	Induction of osteoblast differentiation, inhibition of oxidative stress	2018, [79]
**4**	Ginsenoside Rb2	MC3T3-E1	ALP	10 μM, 48 h	Ovariectomized osteoporosis mice	18.5 μmol/kg, 12 weeks	Increase in osteoblasts viability, induction of osteoblasts differentiation, inhibition of oxidative stress	2014, [80]
**5**	Ginsenoside Rb2	RAW264.7	TRAP	10 μM, 72 h	N/A	N/A	Suppression of osteoclast differentiation, suppression of NF-κB and STAT3 pathways	2017, [81]
**6**	Ginsenoside Rb2	N/A	N/A	N/A	Ketogenic-diet-induced osteoporosis mice	20 mg/kg, 12 weeks	Increase in osteoblast activity, reduction in osteoclast activity	2021, [82]
**7**	Ginsenoside Rc	MC3T3-E1	ALP	200 μM, 14 days	Ovariectomized osteoporosis mice	50 mg/kg, 3 months	Induction of osteoblasts differentiation, activation of Wnt/β-catenin	2022, [83]
**8**	Ginsenoside Rc	Osteoblasts from human skull	ALP	10 μM, N/A	Ovariectomized osteoporosis rats	20 mg/kg, 4 weeks	Induction of osteoblasts differentiation, activation of TGF-β/Smad pathway	2024, [84]
**9**	Ginsenoside Re	MC3T3-E1	ALP	50 μM, 14 days	Zebrafish	50 μM, 5 weeks	Induction of osteoblasts differentiation	2016, [85]
**10**	Ginsenoside Re	Macrophages from mouse bone marrow	TRAP	5 μM, 72 h	Zebrafish	5 μM, 5 weeks	Suppression of osteoclast differentiation, downregulation of NFATc1, c-Fos, and pErk, suppression of NF-κB pathway	2016, [86]
**11**	Ginsenoside Rg1	Mesenchymal stem cells from rat bone marrow	ALP	1 μM, 14 days	Tibial fractured rats	20 mg/kg, 3 weeks	Induction of osteoblasts differentiation, activation of BMP-2/Smad pathway	2016, [87]
**12**	Ginsenoside Rg1	Osteoprogenitors from rat femur	ALP	164.8 uM, 7 days	Goto-Kakizaki type 2 diabete rats	10 mg/kg, 12 weeks	Induction of osteoblasts differentiation, activation of angiogenesis	2022, [88]
**13**	Ginsenoside Rg1	MC3T3-E1	ALP	50 μM, 7 days	Glucocorticoid-induced osteoporosis Zebrafishes	50 μM, 10 days	Induction of osteoblasts differentiation, activation of GPER/PI3K/Akt pathway	2024, [89]
**14**	Ginsenoside Rg3	RAW264.7	TRAP	10 μM, 7 days	N/A	N/A	Suppression of osteoclast differentiation, suppression of NF-κB and MAPK pathways	2015, [90]
**15**	Ginsenoside Rg3	Osteoblasts from rat skull	ALP	10 μM, 14 days	Glucocorticoid-induced osteoporosis rats	20 mg/kg, 5 weeks	Induction of osteoblasts differentiation, activation of BMP-2/BMPR1A/Runx2 pathway	2016, [91]
**16**	Ginsenoside Rg3	N/A	N/A	N/A	AlCl3-induced osteoporosis rats	20 mg/kg, 1 month	Increase in osteoblast growth factors, inhibition of oxidative stress	2020, [92]
**17**	Ginsenoside Rg3	MC3T3-E1	ALP	20 μM, 5 days	Ovariectomized osteoporosis rats	20 mg/kg, 5 weeks	Induction of osteoblasts differentiation, activation of osteoblast autophagy, activation of AMPK pathway, suppression of mTOR pathway	2020, [93]
**18**	Ginsenoside Rg3	MC3T3-E1	ALP	10 μM, 24 h	AlCl3-induced osteoporosis rats	20 mg/kg, 1 month	Induction of osteoblasts differentiation, inhibition of osteoblast apoptosis, activation of TGF-β1/Smad pathway	2021, [94]
**19**	Ginsenosides Rg5 + Ginsenosides Rk1	MC3T3-E1	ALP	20 μg/mL, 12 days	N/A	N/A	Induction of osteoblasts differentiation, activation of BMP-2/Runx2 pathway	2014, [95]
**20**	Ginsenoside Rh1	MC3T3-E1	ALP	10 μM, 12 days	N/A	N/A	Induction of osteoblasts differentiation, inhibition of oxidative stress, activation of BMP-2/Runx2 pathway	2014, [96]
**21**	Ginsenoside CK	Mesenchymal stem cells from rat bone marrow	Alizarin Red S	10 μM, 14 days	Femoral fractured rats	500 μM, 4 weeks	Induction of osteoblasts differentiation, upregulation of β-catenin and Runx2	2022, [97]
**22**	Ginsenoside CK	RAW264.7	TRAP	10 μM, 5 days	Ovariectomized osteoporosis mice	10 mg/kg, 8 weeks	Suppression of osteoclast differentiation, inhibition of oxidative stress, suppression of NF-κB pathway	2023, [98]
**23**	Red ginseng extract	MC3T3-E1	ALP	125 μg/mL, 7 days	Glucocorticoid-induced osteoporosis mice	500 mg/kg, 5 weeks	Induction of osteoblasts differentiation, upregulation of pAkt, downregulation of pJNK	2015, [99]
**24**	Ginseng extract	RAW264.7	TRAP	50 μg/mL, 5 days	Ovariectomized osteoporosis rats	300 mg/kg, 8 weeks	Suppression of osteoclast differentiation	2015, [100]
**25**	Ginseng leaf extract	RAW264.7	TRAP	1 mg/mL, 5 days	N/A	N/A	Suppression of osteoclast differentiation, inhibition of oxidative stress, downregulation of HMGB1, upregulation of Nrf2 and HO-1	2024, [101]
**26**	Ginseng-derived ginsenosides containing nanovesicles	Macrophages from mouse bone marrow	TRAP	1 μg/mL, 4 days	Lipopolysaccharide-induced osteoporosis mice	1 mg/kg, 1 week	Suppression of osteoclast differentiation, suppression of NF-κB and MAPK pathways	2023, [102]

Assay ^a^: Specific kinds of basic assay for the anti-osteoporosis ability. Dose ^b^: Most effective dose of each ginsenoside and extract in cell model assay. ALP: Alkaline phosphatase. TRAP: Tartrate-resistant acid phosphatase. CCK8: Cell counting kit 8. N/A: Not available.

**Table 3:** Phytochemicals from ginseng with anti-osteoarthritis activity.

No.	Phytochemicals	Cell Model	Assay ^a^	Dose ^b^, Time	Animal Model	Dose, Time	Mechanism	Year, Ref.
**1**	Ginsenoside Rb1	SW1353	Quantification of NO	80 μM, 24 h	N/A	N/A	Reduction in NO production, downregulation of iNOS, and nuclear p65	2014, [108]
**2**	Ginsenoside Rb1	SW1353	WB of MMP-13	80 μM, 24 h	ACLT osteoarthritis rats	80 μM, 6 weeks	Upregulation of type II collagen, downregulation of MMP-13, Notch1, and JAG1	2015, [109]
**3**	Ginsenoside Rb1	C5.18	RT-PCR of MMP-13	100 μg/mL, 24 h	ACLT + MCLT osteoarthritis rats	300 μM, 4 weeks	Downregulation of MMP-13 and type X collagen	2016, [110]
**4**	Ginsenoside Rb1	Chondrocytes from each group	RT-PCR of MMP-13	10 μg/kg, 4 weeks	MIA-induced and Ovariectomized osteoarthritis rats	10 μg/kg, 4 weeks	Downregulation of IFN-γ, CCL-2/MCP1, IL-6, IL-1β, MMP-13, Cox-2, TGF-β, CTX-1. and PGE2, upregulation of BMP-2	2021, [111]
**5**	Ginsenoside Rb1	Chondrocytes from healthy rats	CCK8	10 μM, 24 h	MIA-induced osteoarthritis rats	10 mg/kg, 2 weeks	Downregulation of IL-6, IL-1β, TNF-α, and miR-21-5p, upregulation of FGF18	2022, [112]
**6**	Ginsenoside Rb1	N/A	N/A	N/A	Hollow trephine-damaged osteoarthritis rabbits	100 μg/kg, 10 days	Suppression of chondrocyte apoptosis, reduction in ROS production, upregulation of TIMP-1, downregulation of PGE2, MMP-1, MMP-3, MMP-13, TNF-α, Caspase-3, Bax, pAkt, pP65, and pp38	2022, [113]
**7**	Ginsenoside Rg1	Chondrocytes from healthy rats	MTT	10 μg/mL, 72 h	N/A	N/A	Suppression of chondrocyte apoptosis, upregulation of pAkt, Bcl2, and TIMP-1, downregulation of Bax and MMP-13	2014, [114]
**8**	Ginsenoside Rg1	Chondrocytes from osteoarthritis patients	RT-PCR of MMP-13	10 μg/mL, 24 h	ACLT osteoarthritis rats	60 mg/kg, 12 weeks	Upregulation of type II collagen and aggrecan, downregulation of MMP-13 and COX-2	2017, [115]
**9**	Ginsenoside Rg1	Chondrocytes from healthy humans	CCK8	100 μg/mL, 72 h	N/A	N/A	Suppression of chondrocyte apoptosis, reduction in ROS and MDA production, upregulation of Bcl2, downregulation of Bax, Caspase-3, Caspase-8, Caspase-9, FasL, and AIF	2022, [116]
**10**	Ginsenoside Rg1	Chondrocytes from healthy chickens	CCK8	40 μM, 24 h	Thiram-induced osteoarthritis chickens	40 mg/kg, 18 days	Suppression of chondrocyte apoptosis, upregulation of Bcl2, HIF-1α, VEGFA, and VEGFR2, downregulation of Bax and Caspase-3	2023, [117]
**11**	Ginsenoside Rg1	Chondrocytes differentiated from healthy rabbit adipose stem cells	MTT	40 μM, 5 days	Papain-induced osteoarthritis rabbits	1:1 (V:V) mixture of hyaluronic acid and adipose stem cells (2 × 10^7^/mL) suspension in 40 μM Rg1, 9 days	Upregulation of TIMP-1, downregulation of MMP-13	2024, [118]
**12**	Ginsenoside Rg3	SW1353, chondrocytes from healthy rabbit	WB of MMP-13, DMMB	20 μM, 24 h, 2 μM, 3 days	N/A	N/A	Downregulation of MMP-13, inhibition of glycosaminoglycan release	2014, [119]
**13**	Ginsenoside Rg3	TC28a2	WB of SIRT1	20 μM, 24 h	N/A	N/A	Suppression of chondrocyte apoptosis, reduction in ROS production, upregulation of Bcl2, downregulation of IL-8, MMP-13, Bax, and pp38, activation of SIRT1/PGC-1α/SIRT3 pathway, suppression of NF-κB pathway	2021, [120]
**14**	Ginsenosides Rg5	Chondrocytes from healthy rats	TUNEL	15 μM, 48 h	MCLT osteoarthritis rats	15 mg/kg, 1 month	Suppression of chondrocyte apoptosis, reduction in NO production, upregulation of type II collagen and TIMP-1, downregulation of MMP-13, IL-1β, iNOS, and TNF-α	2017, [121]
**15**	Ginsenoside CK	Chondrocytes from osteoarthritis patients	Quantification of NO	5 μM, 24 h	N/A	N/A	Reduction in NO production, downregulation of iNOS, MMP-13, pJNK, pp38, and pErk, inhibition of glycosaminoglycan release	2018, [122]
**16**	Ginsenoside CK	Chondrocytes from healthy mouse	CCK8	50 μM, 48 h	MCLT osteoarthritis mouse	40 mg/kg, 8 weeks	Suppression of chondrocyte pyroptosis, upregulation of type II collagen, downregulation of IL-6, MMP-3, MMP-13, ADAMTS5, NLRP3, GSDMD, cleaved caspase-1, and IL-1β, inhibition of glycosaminoglycan release	2023, [123]
**17**	Ginsenoside CK	Chondrocytes from healthy rats	WB of MMP-13	30 nM, 24 h	MIA-induced osteoarthritis rats	80 mg/kg, 7 weeks	Suppression of chondrocyte pyroptosis, upregulation of type II collagen, downregulation of MMP-3, MMP-13, ADAMTS4, ADAMTS5, NLRP3, GSDMD, cleaved caspase-1, and IL-1β,	2023, [124]

Assay ^a^: Specific kinds of basic assay for the anti-osteoarthritis ability. Dose ^b^: Most effective dose of each ginsenoside in cell model assay. NO: Nitric oxide. ACLT: Anterior cruciate ligament transection. MCLT: Medial cruciate ligament transection. MIA: Monoiodoacetate. CCK8: Cell counting kit 8. DMMB: Dimethylmethylene blue assay. TUNEL: TdT-mediated dUTP-biotin nick end labeling. MTT: 3-(4,5-dimethylthiazol-2-yl)-2,5-diphenyltetrazolium bromide. N/A: Not available.

## Data Availability

The author declares that there are no primary datasets and computer codes associated with this study. All data and materials are available to the researchers once published.
